# Hydrogen sulfide attenuates mitochondrial dysfunction-induced cellular senescence and apoptosis in alveolar epithelial cells by upregulating sirtuin 1

**DOI:** 10.18632/aging.102454

**Published:** 2019-12-23

**Authors:** Ruijuan Guan, Zhou Cai, Jian Wang, Mingjing Ding, Ziying Li, Jingyi Xu, Yuanyuan Li, Jingpei Li, Hongwei Yao, Wei Liu, Jing Qian, Bingxian Deng, Chun Tang, Dejun Sun, Wenju Lu

**Affiliations:** 1 State Key Laboratory of Respiratory Diseases, Guangdong Key Laboratory of Vascular Diseases, National Clinical Research Center for Respiratory Diseases, Guangzhou Institute of Respiratory Health, The First Affiliated Hospital of Guangzhou Medical University, Guangzhou, Guangdong, China; 2 Key Laboratory of National Health Commission for the Diagnosis and Treatment of COPD, Departments of Respiratory and Critical Diseases, Inner Mongolia Autonomous Region People's Hospital, Hohhot, China

**Keywords:** hydrogen sulfide, cigarette smoke extract, alveolar epithelial cell, mitochondria injury, senescence

## Abstract

Hydrogen sulfide (H_2_S), an endogenous gaseous signal molecule, regulates many pathologies related to aging. Sirtuin 1 (SIRT1) has been shown to protect against mitochondrial dysfunction and other pathological processes, including premature senescence. This study was aimed to investigate whether and how H_2_S attenuates senescence and apoptosis of alveolar epithelial cells via a SIRT1-dependent mechanism. Our results showed that treatment with sodium hydrosulfide (NaHS), a donor of H_2_S, attenuated cigarette smoke extract (CSE)-induced oxidative stress, mitochondrial dysfunction, cellular senescence and apoptosis in A549 cells. This was associated with SIRT1 upregulation. SIRT1 activation by a pharmacological activator, SRT1720, attenuated CSE-induced oxidative stress and mitochondrial dysfunction in A549 cells. While SIRT1 inhibition by EX 527 or silencing by siRNA transfection significantly attenuated or abolished the ability of NaHS to reverse the CSE-induced oxidative stress, mitochondrial dysfunction and the imbalance of mitochondrial fusion and fission. Also, SIRT1 inhibition or silencing abolished the protection of NaHS against CSE-induced cellular senescence and apoptosis. In conclusion, H_2_S attenuates CSE-induced cellular senescence and apoptosis by improving mitochondrial function and reducing oxidative stress in alveolar epithelial cells in a SIRT1-dependent manner. These findings provide novel mechanisms underlying the protection of H_2_S against cigarette smoke-induced COPD.

## INTRODUCTION

Cigarette smoke (CS) is one of the most common inhaled irritants of the respiratory tract; it contains approximately 5000 chemical compositions including particles, gases, free radicals and reactive chemicals [1]. CS can induce an oxidant burden on the lungs, which promotes the pathogenesis of lung cancer, pulmonary fibrosis and chronic obstructive pulmonary disease (COPD) [2, 3]. Alveolar epithelial cells (AECs) appear to be a direct target for oxidant injury of the various cell types of the lung. Accelerated cellular senescence and apoptosis, as well as the accumulation of mitochondrial damage, in the alveolar epithelium by CS are considered as essential processes in the pathogenesis of smoking-associated pulmonary diseases, including COPD [4, 5]. Therefore, the protection of AECs from injury by CS appears to be crucial for the management of numerous lung diseases associated with cigarette smoking.

Sirtuin 1 (SIRT1), a nicotinamide adenine dinucleotide (NAD+)-dependent histone deacetylase, was initially regarded as a critical enzyme that increased life expectancy in yeast, worm, fly and mice [6–8]. Recently, SIRT1 has been shown to regulate many physiological and pathophysiological processes, including cellular senescence/aging, apoptosis, stress resistance, metabolism and autoimmunity [9]. Yeung and co-workers found that SIRT1 negatively regulate the RelA/p65 subunit of NF-kB and represses gene transcription via deacetylating RelA/p65 at lysine 310 [10]. SIRT1 also activates a stress-response transcription factor, FOXO3, thereby modulating mitochondria dysfunction, oxidative stress, and cellular senescence [11–13]. Yao and colleagues have shown that SIRT1 is reduced in the lungs of patients with COPD and activation of SIRT1 by a selective pharmacological activator, SRT1720, protects against pulmonary emphysema in mice [12]. Hence, pharmacological interventions that activate SIRT1 signaling in epithelial cells might be beneficial in the prevention and treatment of tissue damage associated with prolonged oxidative stress.

Hydrogen sulfide (H_2_S), a metabolic product of methionine, is synthesized from L-cysteine primarily by three key enzymes: cystathionine-c-lyase (CGL), cystathionine-b-synthetase (CBS) and 3-mercaptypyruvate sulfurtransferase (MPST) [14]. Identified as the third gasotransmitter, along with nitric oxide and carbon monoxide, H_2_S modulates a variety of physiological functions including anti-oxidative stress, anti-senescence/aging and anti-apoptotic effects [15–17]. Kumar and colleagues showed that exogenous H_2_S mitigates homocysteine-induced neurotoxicity via preventing mitochondrial dysfunctions and oxidative damage [18]. Zheng et al demonstrated that H_2_S prevents nicotinamide-induced premature senescence in human umbilical vein endothelial cells via upregulation of SIRT1 [19]. H_2_S also protects against hydrogen peroxide-induced apoptosis through the SIRT1 pathway in H9c2 cardiomyocytes [20]. The latest study confirmed that H_2_S content is reduced in the lungs of smokers and COPD patients [21], and H_2_S supplementation attenuates nicotine-induced endoplasmic reticulum stress and apoptosis in bronchial epithelial 16HBE cells [22]. However, to our knowledge, it is still not clear whether or not H_2_S can attenuate oxidative stress and mitochondrial dysfunction-induced cellular senescence and apoptosis in cigarette smoke extract (CSE)-exposed alveolar epithelial cells, or if the protective effect of H_2_S is correlated with SIRT1 pathway.

In this study, we demonstrated that H_2_S donor NaHS significantly inhibited CSE-induced mitochondrial dysfunction, oxidative damage, cell senescence and apoptosis in alveolar epithelial A549 cells. Moreover, the protective effects of NaHS were associated with the increase of CSE-induced SIRT1 expression. These findings provide novel mechanistic evidence of H_2_S in preventing the development of COPD.

## RESULTS

### Effects of NaHS on cell viability, apoptosis and senescence in CSE-stimulated A549 cells

**Figure 1:**
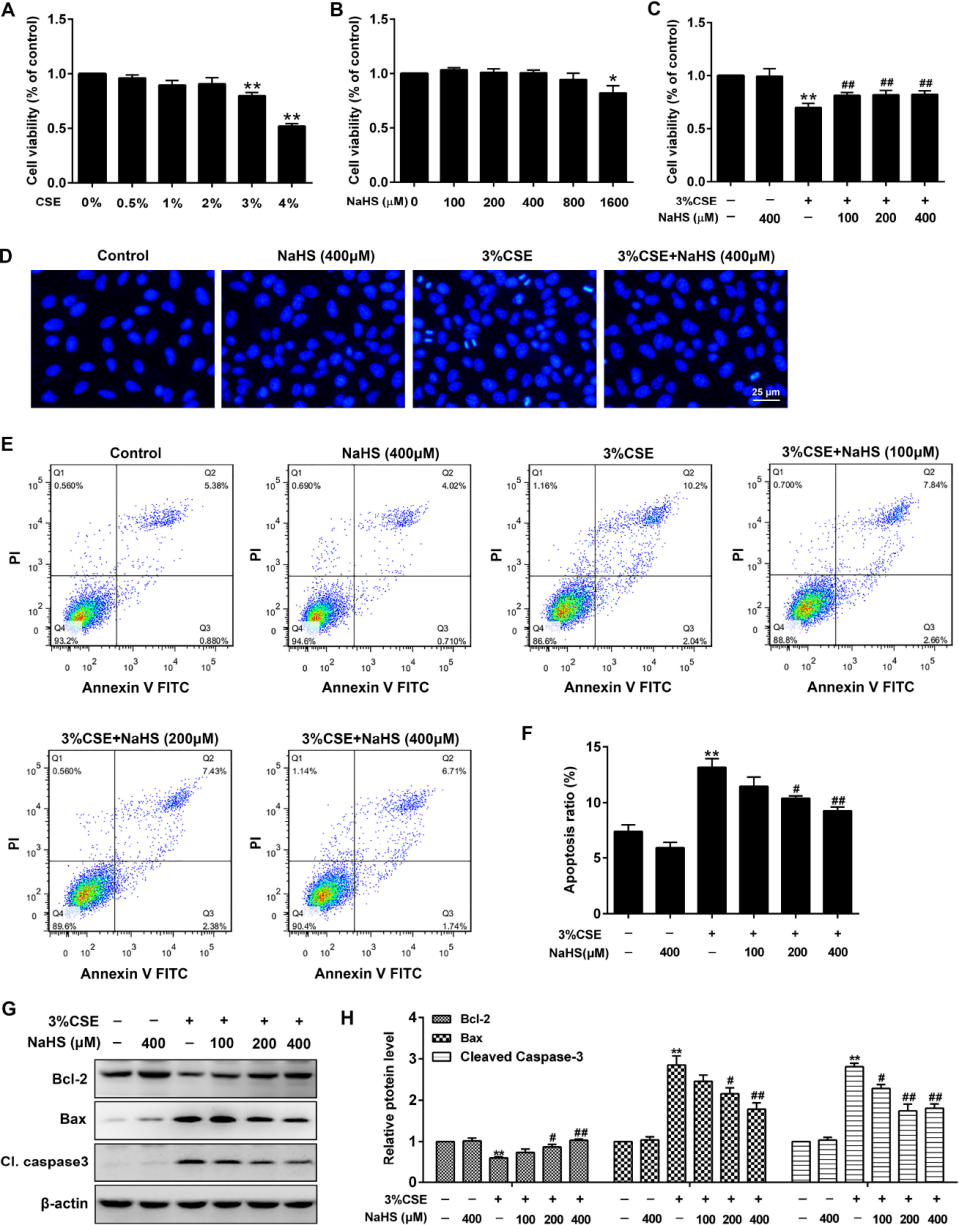
**Effects of NaHS on cell viability and apoptosis in CSE-stimulated A549 cells.** (**A**, **B**) A549 cells were treated with different doses of CSE or NaHS for 48h. The cells stimulated with vehicle only served as controls. Cell viability was detected by CCK-8 assay. ^*^P<0.05, ^**^P<0.01, significantly different from control cells. A549 cells were cultured with and without 3% CSE and/or 100, 200, or 400μM NaHS for 48 h. (**C**) Cell viability of A549 cells with different treatments was measured by CCK-8 assay. (**D**) A549 cells were stained with Hoechst 33258 after treating with and without 3% CSE and/or 400μM NaHS for 48 h, and were examined under the fluorescence microscopy. (**E**) The cells were double-stained with Annexin V-FITC and PI, and then the cellular apoptosis was determined by flow cytometry. (**F**) The ratio of apoptotic cells was statistically analyzed. (**G**, **H**) The protein levels of Bcl-2, Bax and Cleaved caspase 3 were detected using Western blot. ^**^P<0.01, significantly different from control cells [3% CSE (-) and NaHS (-)]; ^#^*P*<0.05, ^##^*P*<0.01, significantly different from cells treated with 3% CSE only.

A CCK-8 assay was performed to determine the effective dose of CSE on A549 cells for 48 h exposure. As shown in Figure 1A, more than 3% CSE significantly reduced cell viability of A549 cells. To investigate the role of NaHS in alleviating CSE-induced cell damage, A549 cells were exposed to different doses of NaHS with or without CSE presence. As shown in Figure 1C, after a 48 h NaHS treatment, the CSE-caused cell damage in A549 cells was largely abolished. However, there were no discernible dose-response differences between NaHS (100, 200 or 400 μM)-treated cells and control cells (Figure 1B). Hoechst staining was employed to confirm the cell damage induced by CSE. Cells with CSE exposure contained small bright blue dots representing apoptotic cells, which were significantly reduced by NaHS (400 μM) (Figure 1D). Likewise, flow cytometry assay also showed that treatment with NaHS reduced CSE-induced cell apoptosis in A549 cells in a dose-dependent manner (Figure 1E, 1F). Treatment with NaHS also increased the expression of anti-apoptotic protein Bcl-2 but reduced the pro-apoptotic proteins Bax and Cleaved caspase 3 (Figure 1G, 1H). Therefore, the Bcl-2/Bax ratio was significantly increased after NaHS intervention (Supplementary Figure 1). These results suggest the inhibitory effects of NaHS on alveolar epithelial cell apoptosis.

**Figure 2:**
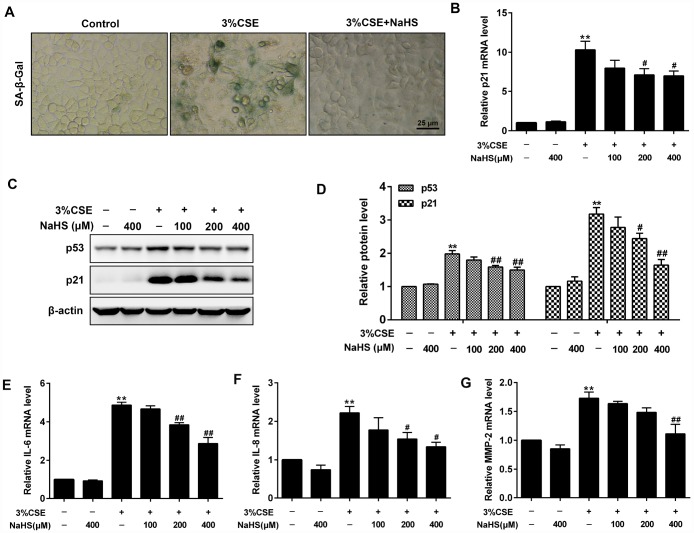
**Effects of NaHS on cell senescence in CSE-stimulated A549 cells.** A549 cells were cultured with and without 3% CSE and/or 100, 200, or 400μM NaHS for 48 h. Cell senescence was performed by examining the (**A**) the SA–β-gal activity. (**B**) the mRNA level of p21 by Real-time PCR. (**C**, **D**) the protein levels of p53 and p21 by Western blot. (**E**–**G**) The mRNA levels of IL-6, IL-8 and MMP-2 were detected using Real-time PCR. ^**^P<0.01, significantly different from control cells [3%CSE (-) and NaHS (-)]; ^#^*P*<0.05, ^##^*P*<0.01, significantly different from cells treated with 3%CSE only.

In addition, β-Galactosidase staining and Western blot assays were performed to determine cell senescence induced by CSE. As expected, CSE exposure significantly increased senescence-associated β-gal (SA–β-gal) activity and the levels of pro-senescent proteins (i.e., p21 and p53) in A549 cells. These effects were significantly reduced after NaHS treatment (Figure 2A–2D). Senescence cells are metabolically active and thus secrete some inflammatory mediators such as IL-8, IL-6 and MMPs, which is called senescence-associated secretory phenotype (SASP) [23]. NaHS treatment also inhibited CSE-induced upregulation of SASP, as demonstrated by decreased IL-6, IL-8 and MMP-2 levels (Figure 2E–2G). Altogether, these results suggest that NaHS attenuates CSE-induced apoptosis and senescence in human alveolar epithelial A549 cells.

### Effects of NaHS on CSE-induced oxidative stress in A549 cells

**Figure 3:**
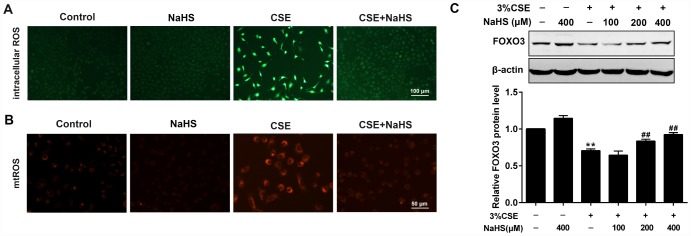
**Effects of NaHS on CSE-induced oxidative stress in A549 cells.** A549 cells treated with and without 3% CSE and/or 400μM NaHS for 48 h. Representative microphotographs showing intracellular ROS (**A**) and mtROS (**B**) generation respectively. (**C**) A549 cells were cultured with and without 3% CSE and/or 100, 200, or 400μM NaHS for 48 h. Western blot was used to analyze the protein expression of FOXO3. ^**^P<0.01, significantly different from control cells [3%CSE (-) and NaHS (-)];^##^*P*<0.01, significantly different from cells treated with 3%CSE only.

CS contains exogenous reactive oxygen species (ROS) and also causes the release of endogenous ROS, which has been identified as an explanation behind CSE toxicity. We then investigated the effects of NaHS on oxidative stress. Fluorescence microscopy showed that NaHS treatment significantly reduced the intracellular ROS levels stimulated by CSE (Figure 3A). CSE exposure for 48 h also increased mitochondrial ROS (mtROS) levels in A549 cells, which was also significantly inhibited by NaHS treatment (Figure 3B). Additionally, FOXO3 is a transcription factor whose activation results in the inhibition of ROS generation. In our experiments, NaHS also increased FOXO3 expression in CSE-stimulated A549 cells (Figure 3C). These above data suggest that NaHS significantly reduced CSE-induced oxidative stress in A549 cells.

### Effects of NaHS on mitochondrial function in CSE-stimulated A549 cells

**Figure 4:**
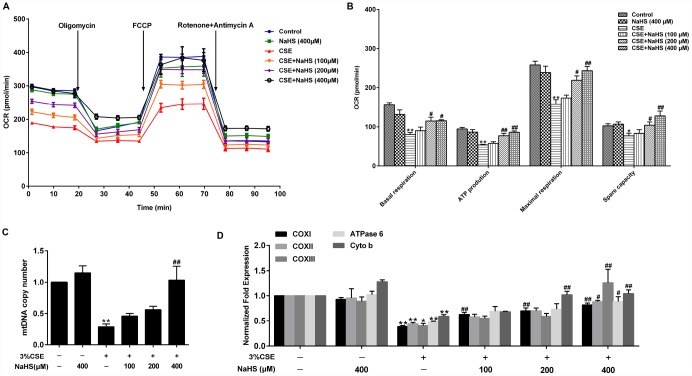
**Effects of NaHS on mitochondrial function in CSE-stimulated A549 cells.** A549 cells were cultured with and without 3% CSE and/or 100, 200, or 400μM NaHS for 48 h. (**A**) The bioenergetic profiles of A549 cells were measured by a Seahorse Extracellular Flux Analyzer, OCR in cells treated with oligomycin, FCCP, and rotenone and Antimycin A. (**B**) Quantitative analysis of basal respiration, ATP production, maximal respiratory and spare capacity is shown. (**C**) mtDNA copy number was measured by Real-time PCR. (**D**) The mRNA levels of COXI, COXII, COXIII, ATPase 6 and Cyto b were detected using Real-time PCR. ^*^P<0.05, ^**^P<0.01, significantly different from control cells [3%CSE (-) and NaHS (-)]; ^#^*P*<0.05, ^##^*P*<0.01, significantly different from cells treated with 3%CSE only.

Mitochondria are an important source of ROS within most mammalian cells. We then took a further step to examine the effect of NaHS on mitochondrial function by detecting oxygen consumption rate (OCR) in A549 cells with CSE stimulation. Basal respiration, ATP production, maximal respiration and spare respiratory capacity were significantly reduced in CSE-stimulated A549 cells, all of which were improved by NaHS (Figure 4A, 4B). Mitochondrial DNA (mtDNA) is a sensitive indicator of mitochondrial function. Our results showed that the mtDNA copy number in the CSE-treated A549 cells was also significantly lower than that in the control cells and was increased in the cells treated with NaHS (Figure 4C). We also determined the alterations of mtDNA-encoded genes, which play an important role in mitochondrial function. The results showed that the transcription levels of mtDNA-encoded subunits, including COXI, COXII, COXIII, ATPase 6 and Cyto b, were significantly decreased in CSE-stimulated cells compared with those in control cells, and these effects were increased by NaHS treatment (Figure 4D). These data indicate that NaHS prevents CSE-induced mitochondrial dysfunction in A549 cells.

### Effects of NaHS on CSE-induced reduction of SIRT1 in alveolar epithelial A549 cells

**Figure 5:**
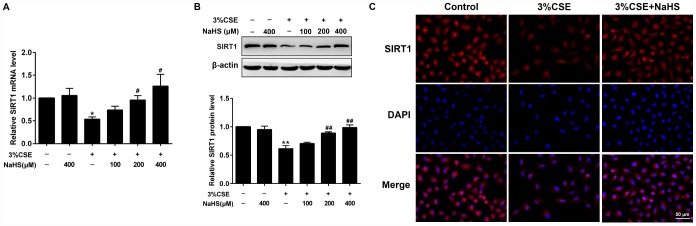
**Effects of NaHS on SIRT1 mRNA and protein expressions in CSE-stimulated epithelial A549 cells.** A549 cells were cultured with and without 3% CSE and/or 100, 200, or 400μM NaHS for 48 h. (**A**) The mRNA level of SIRT1 was detected using Real-time PCR. (**B**) The protein level of SIRT1 was detected using Western blot. (**C**) Immunofluorescence staining for SIRT1 was performed on A549 cells treated with and without 3% CSE and 400μM NaHS for 48 h. ^*^P<0.05, ^**^P<0.01, significantly different from control cells [3%CSE (-) and NaHS (-)]; ^#^*P*<0.05, ^##^*P*<0.01, significantly different from cells treated with 3%CSE only.

SIRT1 has been shown to regulate apoptosis, senescence, oxidative stress, and mitochondrial dysfunction. Henceforth we determined SIRT1 gene expression and protein levels in the absence or presence of NaHS treatment in response to CSE stimulation. Real-time PCR and Western blot results showed that the mRNA and protein levels of SIRT1 were remarkably reduced in CSE-stimulated A549 cells. Expression of SIRT1 levels was restored to near or even higher than baseline by NaHS treatment (Figure 5A, 5B). Similarly, our immunofluorescence studies showed that the fluorescence intensity of SIRT1 also increased with NaHS treatment (Figure 5C). These results demonstrate that NaHS increases SIRT1 transcription and expression in alveolar epithelial A549 cells induced by CSE, and suggest that SIRT1 is involved in the protective effect of NaHS on cellular senescence and apoptosis.

### Effects of SIRT1 on the NaHS-mediated reduction in the CSE-induced oxidative stress in A549 cells

**Figure 6:**
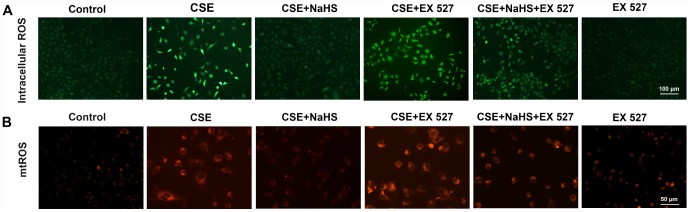
**Effects of SIRT1 on the NaHS-mediated reduction in the oxidative stress in CSE-stimulated A549 cells.** A549 cells were cultured with SIRT1 inhibitor (EX 527) in the absence and presence of 3% CSE and NaHS for 48 h. (**A**) Generation of intracellular ROS was determined by the ROS Assay Kit. (**B**) Generation of mtROS was determined by the MitoSOX^TM^ Red Assay Kit.

To investigate the involvement of SIRT1 in the protective effect of NaHS in alveolar epithelial cells, we first used a selective SIRT1 inhibitor, EX 527, to inhibit SIRT1. As shown in Figure 6, when SIRT1 was suppressed by EX 527, the protection of NaHS against CSE-induced oxidative stress was abolished or largely diminished, as indicated by increased intracellular and mitochondrial ROS production (Figure 6). These effects were also blunted by SIRT1 silencing (Supplementary Figure 2). Moreover, the role of SIRT1 in these changes was further confirmed by the observation that the upregulation of SIRT1 by SRT1720, a selective pharmacological SIRT1 activator, induced the opposite changes (Supplementary Figure 3). These results suggest that SIRT1 is involved in the protection of NaHS against CSE-induced oxidative stress.

### Effects of SIRT1 on the NaHS-mediated attenuation of mitochondrial dysfunction in CSE-exposed A549 cells

**Figure 7:**
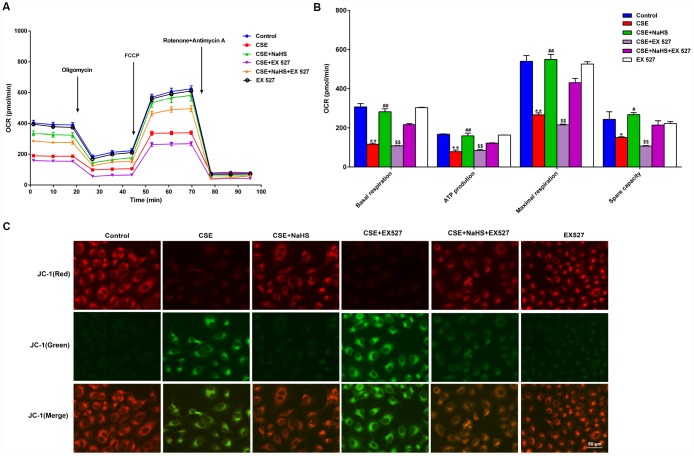
**Effects of SIRT1 on the NaHS-mediated mitochondrial damage in CSE-stimulated A549 cells.** A549 cells were cultured with SIRT1 inhibitor (EX527) in the absence and presence of 3% CSE and NaHS for 48 h. (**A**) The bioenergetic profiles of A549 cells were measured by a Seahorse Extracellular Flux Analyzer, OCR in cells treated with oligomycin, FCCP, and rotenone and Antimycin A. (**B**) Quantitative analysis of basal respiration, ATP production, maximal respiratory and spare capacity is shown. (**C**) Mitochondrial permeability potential was determined by JC-1 staining. Red fluorescence represented normal membrane potential, and green fluorescence represented mitochondrial membrane potential depolarization. ^*^P<0.05, ^**^P<0.01, significantly different from control cells [CSE (-), NaHS (-) and EX 527 (-)]; ^#^*P*<0.05, ^##^*P*<0.01, significantly different from cells treated with 3% CSE only; ^&&^*P*<0.01, significantly different from cells treated with EX 527 only.

To determine whether SIRT1 participates in NaHS-mediated attenuation of CSE-induced mitochondrial dysfunction, we next tested the effect of NaHS on OCR in A549 cells with CSE stimulation and SIRT1 inhibition. Consistent with the above data, treatment with NaHS (400 μM) increased the CSE-induced downregulation of the basal respiration, ATP production, spare respiratory capacity and maximal respiration in A549 cells. However, when SIRT1 was suppressed by EX 527 treatment, the protection of NaHS against CSE-induced mitochondrial dysfunction was partially reversed (Figure 7A, 7B). Interestingly, EX 527 treatment alone had no effects on OCR regardless of CSE treatment. Likewise, when SIRT1 was silenced by SIRT1-specific siRNA, these bioenergetics parameters were no longer able to be improved by NaHS (Supplementary Figure 4). In contrast, SIRT1 activation by SRT1720 significantly inhibited CSE-induced mitochondrial dysfunction (Supplementary Figure 5).

Mitochondrial membrane potential (Δψm) was determined by JC-1 staining. Normal membrane potential exhibited red fluorescence intensity, while depolarization of the Δψm showed green fluorescence intensity. CSE enhanced green fluorescence intensity but reduced red fluorescence intensity, implying that the Δψm of these cells was significantly decreased. Treatment with NaHS decreased green fluorescence intensity but increased red fluorescence intensity, indicating that NaHS attenuated the collapse of Δψm in CSE-stimulated A549 cells. However, when SIRT1 was inhibited, these above protective effects on Δψm were blunted (Figure 7C). Therefore, NaHS may improve mitochondrial function in CSE-stimulated alveolar epithelial cells via SIRT1 signaling.

### Effects of SIRT1 on the NaHS-mediated alteration of mitochondrial morphology and mitochondrial dynamics-related protein expression in CSE-exposed A549 cells

**Figure 8:**
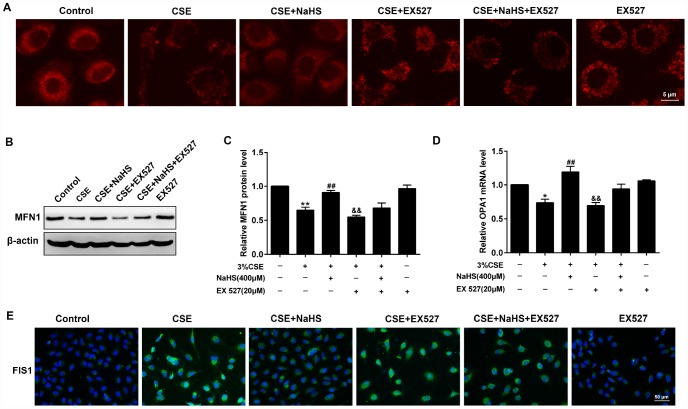
**Effects of SIRT1 on the NaHS-mediated the alteration of mitochondrial morphology and mitochondrial dynamics-related protein expression in CSE-stimulated A549 cells.** A549 cells were cultured with SIRT1 inhibitor (EX 527) in the absence and presence of 3% CSE and NaHS for 48 h. (**A**) Representative images for visualization of the mitochondrial morphology in A549 cells. (**B**, **C**) Western blot was used to analyze the protein level of MFN1. (**D**) Real-time PCR was performed to examine the mRNA level of OPA1. (**E**) Immunofluorescence staining of FIS1 was performed. ^*^P<0.05, ^**^P<0.01, significantly different from control cells [3% CSE (-), NaHS (-) and EX 527 (-)]; ^##^*P*<0.01, significantly different from cells treated with 3% CSE only; ^&&^*P*<0.01, significantly different from cells treated with EX 527 only.

For the morphological analysis of mitochondria, A549 cells were labeled with MitoTracker probes. As shown in Figure 8A, the mitochondria of the control group were filamentous and exhibited a thread-like appearance. CSE incubation resulted in punctate and highly fragmented mitochondria, whereas NaHS treatment attenuated these abnormal morphological changes. To further investigate the mechanism of these changes in mitochondrial function, we measured the expression of mitochondrial fission and fusion proteins that modulate mitochondrial function. The results showed that CSE exposure for 48 h decreased the expression of the mitochondrial fusion proteins MFN1 and OPA1, and enhanced the expression of the mitochondrial fission protein FIS1. Treatment with NaHS (400 μM) significantly upregulated CSE-suppressed MFN1 and OPA1 proteins, and downregulated CSE-induced FIS1 protein level, all of which were abolished by SIRT1 inhibition (Figure 8B–8E). These results suggest that SIRT1 is involved in NaHS’ protection against CSE-induced mitochondrial morphology and dynamics.

### Effects of SIRT1 on the NaHS-mediated cellular senescence and apoptosis in CSE-exposed A549 cells

**Figure 9:**
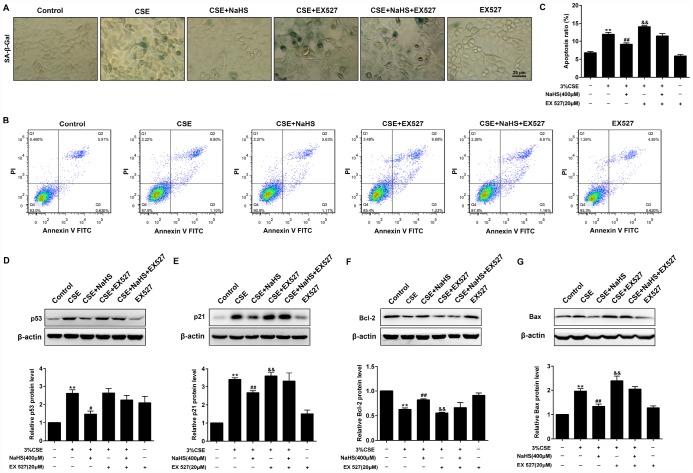
**Effects of SIRT1 on the NaHS-mediated cellular senescence and apoptosis in CSE-stimulated A549 cells.** A549 cells were cultured with SIRT1 inhibitor (EX527) in the absence and presence of 3% CSE and NaHS for 48 h. (**A**) Cell senescence was performed by examining the the SA–β-gal activity. (**B**) The cells were double-stained with Annexin V-FITC and PI, and then the cellular apoptosis was determined by flow cytometry. (**C**) The ratio of apoptotic cells was statistically analyzed. (**D**–**G**) Western blot was used to analyze the protein levels of p53, p21, Bcl-2 and Bax. ^**^P<0.01, significantly different from control cells [3%CSE (-), NaHS (-) and EX 527 (-)]; ^#^*P*<0.05, ^##^*P*<0.01, significantly different from cells treated with 3%CSE only; ^&^*P*<0.05, ^&&^*P*<0.01, significantly different from cells treated with EX 527 only.

As shown in Figure 9, when SIRT1 was inhibited by EX 527, the SA–β-gal activity (Figure 9A) and expressions of pro-senescent proteins (p21 and p53) (Figure 9D, 9E) were no longer able to be decreased by NaHS. Moreover, EX 527 treatment also abolished the inhibitory effect of NaHS on cell apoptosis (Figure 9B, 9C) and the expression of apoptosis-related proteins Bax and Bcl-2 (Figure 9F, 9G). Furthermore, the silencing of SIRT1 also prevented NaHS from attenuating CSE-induced cellular senescence and apoptosis (Supplementary Figure 6). Together, these results indicated that NaHS attenuated oxidative stress and mitochondrial injury-induced cellular senescence and apoptosis in CSE-stimulated epithelial cells via a SIRT1-dependent manner.

## DISCUSSION

In the present study, we demonstrated that CSE induced oxidative stress, mitochondrial damage, senescence, and apoptosis in alveolar epithelial cells. In A549 cells, CSE induced ROS generation, caused mitochondrial morphology alteration and mitochondrial dysfunction. CSE exposure also caused apoptosis and senescence. These effects were significantly attenuated by treatment of NaHS, an H_2_S donor. We also found that the protective effect of H_2_S on these effects was associated with the upregulation of SIRT1.

Alveolar epithelial cell apoptosis/death response ascribable to ongoing cellular injury/damage has long been considered as a critical element in the COPD pathogenesis, and it was recently confirmed in the lungs of animals and patients with COPD [4]. H_2_S, a gaseous molecule, is identified as the third endogenous gasotransmitter after nitric oxide and carbon monoxide [24]. In this study, we demonstrated that treatment with NaHS significantly increased cell viability and decreased cell apoptosis in CSE-exposed A549 cells, which is in agreement with previous observations on the activity of H_2_S in various cells [15, 25, 18]. However, we noticed that the role of H_2_S in cell viability is sophisticated, with both pro- and anti-apoptotic effects. For example, overproduction of H_2_S induces apoptosis of human aorta smooth muscle cells (HASMC), INS-1E cells, pancreatic acinar cells, and some other type cells [26]. These contradictory results showed that different types of cells might have different reactions to H_2_S, and further suggest that an appropriate concentration of H_2_S reduces apoptotic responses, while higher concentration of H_2_S causes increased apoptosis, even necrosis.

Accelerated aging/cellular senescence is also considered to be a degenerative process caused by accumulated injury/damage that leads to cellular dysfunction, organ damage/failure, and even death [27]. Emphysema (an important component of COPD) develops because of accelerated premature aging of the lung due to alveolar epithelial cellular senescence caused by cigarette smoke and noxious gases [12, 28, 29]. Several studies have suggested that H_2_S is implicated in several diseases including pathologies related to aging [17]. H_2_S alleviates endothelial senescence by selective induction of SRSF2 and HNRNPD [30]. H_2_S also ameliorates postharvest senescence via the regulation of antioxidant defenses [31]. Consistent with these findings, our results showed that NaHS treatment also reduced CSE-induced cellular senescence in alveolar epithelial A549 cells.

Oxidative stress is a crucial mechanism underlying CSE-induced cellular senescence and apoptosis [32]. Numerous studies have shown that H_2_S exerts pulmonary protective effects through its antioxidant properties. For instance, H_2_S can reduce the formation of ROS and NOX2 in the lung after acute lung injury [33]. H_2_S protected hypoxia-induced human bronchial epithelial cells from injury via attenuation of ROS-mediated Ca^2+^ overload and mitochondrial dysfunction [34]. Moreover, a recent study found that H_2_S suppressed oxidative stress in CS-exposed mouse lungs, manifesting as increased GSH/GSSG ratio and decreased 8-OHdG level [24]. Consistent with these previous findings, NaHS also protected against CSE-induced oxidative damage in alveolar epithelial cells, which was mediated via the reduction in intracellular and mitochondrial ROS production. Hence, the protective role of H_2_S in alveolar epithelial cells may partially attribute to the inhibition of oxidative stress.

Mitochondria are critical players in ROS generation, and mitochondrial dysfunction can cause increased oxidative stress leading to senescence and apoptosis of cells [35, 36]. Mitochondria themselves, in turn, are prone to oxidative damage, resulting in further mitochondrial dysfunction. The pathogenic role of mitochondrial dysfunction has increasingly been confirmed in a variety of human diseases including Parkinson’s, Alzheimer’s, cardiac dysfunction, pulmonary disorders as well as aging [37]. Cumulative evidence suggests that CS exposure can cause impaired mitochondrial morphology and function in the epithelium, finally leading to COPD [38, 39]. Our results showed that treatment with NaHS significantly relieved mitochondrial morphology damage and improved mitochondrial function in CSE-stimulated A549 cells. This correlates well with a previous finding that H_2_S exerts cardiac mitochondrial protection in primary cardiomyocytes [14] and reveals that the attenuation of emphysema by H_2_S was mediated, at least in part, through the suppression of CSE-induced mitochondrial dysfunction in alveolar epithelial cells.

Mitochondria are double membrane subcellular organelles whose structure and function are modulated by the fusion/fission balance [38]. Fission/fusion protein mismatch leads to mitochondrial dysfunction, permeabilization of the outer mitochondrial membrane, the release of apoptotic proteins and finally contributes to cell apoptosis/death. MFN1 and OPA1 are the major proteins required for mitochondria fusion. MFN1 deficiency decreases mitochondrial fusion levels, induces fusion imbalance and even aggravates mitochondrial fragmentation [14]. OPA1 mediates the fusion of the inner membrane following MFN-mediated outer membrane fusion [39]. The interaction of DRP1 and FIS1 regulates mitochondrial fission. DRP1 affects the mitochondrial shape, participates in mitochondrial fission, and has been thought of as a therapeutic target in COPD [14, 40]. FIS1, acting as a receptor for DRP1, also promotes mitochondrial fission [41]. Our data also showed that CSE exposure also devastated the balance of mitochondrial fission and fusion protein expression in A549 cells, which is in conformity with previous studies [42]. Whereas treatment with NaHS significantly reduced CSE-induced alterations in the expression of mitochondrial fission/fusion proteins. Thus, H_2_S might improve mitochondrial morphology and function through the orchestration of mitochondrial dynamics.

We further investigated the mechanisms through which H_2_S attenuated CSE-induced mitochondrial dysfunction and oxidative stress. Accumulative evidences have indicated the reduced level and activity of SIRT1 in several types of cells *in vitro* and mouse lungs *in vivo* exposed to cigarette smoke as well as in the lungs of patients with COPD [12, 29, 43, 44]. SRT1720 treatment could enhance the antioxidant genes and enzymes, and thus protected against CS-induced lung oxidative stress via a FOXO3-dependent mechanism [12]. Deficiency of SIRT1 weakened mitochondrial function and increased oxidative stress in the lung. Also, decreased nuclear NAD^+^ and diminished SIRT1 activity underlaid a specific loss of mitochondrial-encoded subunits of the oxidative phosphorylation system. These findings suggest that SIRT1 is crucial to maintain mitochondrial environmental stability and improve oxidative stress status [45]. In this study, we found that NaHS treatment attenuated CSE-induced reduction of SIRT1, and EX 527 treatment or SIRT1 silencing abolished the protection of NaHS against CSE-induced mitochondrial dysfunction and oxidative stress. Inversely, SIRT1 activation not only restored mitochondrial function, but also strengthened the protective effects of NaHS on mitochondrial function. Taken together, these data suggest that H_2_S protects against CSE-induced mitochondrial dysfunction and oxidative stress via activation of SIRT1.

Given the role of mitochondrial dysfunction and oxidative stress in the physiopathology of cell cellular senescence and apoptosis, we also investigated whether H_2_S protects against CSE-induced cellular senescence and apoptosis via upregulation of SIRT1. It was previously reported that oxidant stress-mediated reduction of SIRT1 caused the loss of its control on target proteins including p53 and FOXO3, thereby promoting the prosenescent and apoptotic responses [46]. SIRT1 protected against emphysema via FOXO3-mediated reduction of premature senescence and apoptosis in mice [12]. Consistent with these observations, our results showed that the protective effects of NaHS on CSE-induced cellular senescence and apoptosis in alveolar epithelial cells was abolished by EX 527 treatment or SIRT1 silencing. This is in agreement with previous findings, which revealed the protective effects of H_2_S in H9c2 cardiomyocytes and human umbilical vein endothelial cells [20, 47]. These findings further suggest that SIRT1 activation mediated the H_2_S-induced effects on CSE-induced cellular senescence and apoptosis in A549 cells.

However, there are some limitations in our investigation. First, although the level of MPST, a critical enzyme generating H_2_S, has been examined (Supplementary Figure 7), due to technical limitation in our laboratory, the physiological and pathological H_2_S level in alveolar epithelial cells has not yet been assessed in this study, which warrants further studies. Second, other factors such as inflammation and endoplasmic reticulum stress that are involved in epithelial cell injury was not investigated in this study. Third, SIRT1 can regulate various cellular processes, including cellular growth, proliferation, and differentiation. This remains to be investigated in terms of NaHS treatment in future study.

In conclusion, our present results demonstrated that H_2_S protects against CSE-induced mitochondrial dysfunction, apoptosis and cell senescence in alveolar epithelial cells, which is associated with SIRT1 upregulation. Our study provides novel molecular mechanisms underlying the protection of NaHS against premature lung aging and development of COPD.

## MATERIALS AND METHODS

### Chemicals and reagents

NaHS was purchased from Sigma-Aldrich (St Louis, MO, USA), and the cigarettes were purchased from Guangdong Tobacco Industry Co., Ltd. (Guangzhou, China). SRT1720 and EX 527 were purchased from Selleck Chemicals (Houston, TX, USA). The TRIzol Reagent was purchased from Ambion (Life Technologies, CA, USA). The PrimeScript RT reagent Kit with gDNA Eraser was from Takara Bio Inc. (Takara, Shiga, Japan), and the SsoFast EvaGreen Supermix was obtained from Bio-Rad Laboratories, Inc. (CA, USA). The primary and second antibodies described in this study include: anti-Bcl-2 and anti-β-actin polyclonal antibodies were purchased from Proteintech (Chicago, IL, USA); anti-MFN1, anti-SIRT1, anti-FOXO3 and anti-Bax antibodies were purchased from Abclonal (Wuhan, China); anti-p21 and anti-p53 antibodies were purchased from Cell Signaling Technology (CA, USA); anti-FIS1 antibodies, and the horseradish peroxidase (HRP)-labeled Goat Anti-Rabbit/Mouse IgG (H+L) were purchased from Abcam Biotechnology (Cambridge, MA, USA). The poly-vinylidene fluoride (PVDF) membranes were from Millipore Corporation (Billerica, MA, USA). ECL-Plus detection kit probed was purchased from Tanon Science and Technology Co., Ltd. (Shanghai, China). Other reagents were all purchased from GBCBIO Technologies Inc. (Guangzhou, China) unless otherwise indicated.

### Cell culture

Human epithelial A549 cells were obtained from the Cell Bank of the Chinese Academy of Sciences (Shanghai, China), and cultured in Dulbecco’s modified Eagle’s medium (DMEM) (Gibco, NY, USA) supplemented with 10% fetal bovine serum (FBS) (Biochrom, Berlin, Germany) and antibiotics (100 KU/L penicillin and 100 mg/L streptomycin) at 37°C with 5% CO_2_.

### Preparation of CSE

CSE was freshly prepared from two burning cigarettes (Red Roses Label) within 30 min prior to treatments according to a previously described protocol [48].

### Cell viability assay

A549 cell viability was measured according to the manufacturer’s instructions of CCK-8 Kit (Dojindo, Kumamoto, Japan). A549 cells (1×10^4^/well) were seeded in a 96-well plate with DMEM containing 1% FBS. After culture overnight, cells were treated with NaHS and 3% CSE as indicated. The medium was discarded and cells were incubated in 100 μL medium with 10 μL CCK-8 solution at 37°C for 2 h. The absorbance of wells was then measured at 450 nm by a micro-plate reader (Termo Scientifc, Waltham, MA, USA).

### Apoptotic cell determination

A549 cells were plated in a six-well plate (2×10^5^ cells/well) and treated with NaHS in the presence and absence of 3% CSE for 48 h. The cellular apoptotic rate was examined using Annexin V, FITC Apoptosis Detection Kit (R&D Systems, Minneapolis, MN, USA).

### Immunofluorescence staining

Immunofluorescence analysis was performed using a method described previously [49]. Briefly, A549 cells were seeded on glass slides in 12-well plates and incubated as indicated for 48 h. The slides were washed three times with PBS, fixed in 4% paraformaldehyde for 10 min and then permeabilized with 0.1% Triton X-100 for 20 min. Subsequently, the cells were blocked with 3% BSA at room temperature for 1 h and then incubated with primary antibody (SIRT1, 1:100; or FIS1, 1:100) at 4°C overnight. After incubation, slices were washed three times with PBS and incubated with goat anti-mouse FITC-conjugated or goat anti-rabbit Cy3-conjugated second antibody (1:200, Beyotime Institute of Biotechnology, Haimen, China) for 1 h and labeled with DAPI for 5 min. Finally, samples were washed four times with PBS and mounted in antifade mounting medium, and fluorescence was detected using a fluorescence microscope (Olympus, BX53, Japan).

### Measurement of intracellular ROS

Intracellular ROS production was determined by staining A549 cells with an oxidation-sensitive fluorescent probe 2,7-dichlorodihydrofluorescein diacetate (DCF-DA) according to the manufacturer’s protocol. Briefly, A549 cells were treated as indicated for 48 h. Subsequently, the medium was removed, and the cells were incubated with DFCH-DA (Beyotime Institute of Biotechnology, Haimen, China) at a final concentration of 10 μM at 37°C for 20 min. Afterwards, the cells were washed with buffer solution three times as per instructions. The fluorescent intensity was photographed and analyzed with a fluorescence microscope (EVOS^TM^ Auto 2, Invitrogen, WA, USA).

### Measurement of mtROS

mtROS activity was detected with MitoSOX^TM^ Red (ThermoFisher Scientific, MA, USA) assay, a redox-sensitive fluorescent probe that is selectively targeted to the mitochondria. Briefly, A549 cells were seeded in 12-well black plates and treated as indicated. Cells were incubated with 5μmol/L MitoSOX^TM^ reagent for 10 min at 37°C. The cells were washed three times with warm serum-free DMEM, and analyzed for mitochondrial fluorescence using by a fluorescence microscope.

### Mitochondrial respiration assessment

A549 cells (about 10^4^ cells/well) were plated in an XF24-well plate (Seahorse Bioscience, Massachusetts, USA) and treated as indicated. Immediately before the respiration assay, the medium was changed to bicarbonate-free and low-buffered assay medium containing glucose (10mM), pyruvate (1mM) and L-Glutamine (2mM; Life Technologies). OCR was measured by an XF24 Extracellular Flux Analyzer (Seahorse Bioscience) according to the procedure described by the Kit. Firstly, basal respiration was measured in an unbuffered medium. Then oligomycin (1μM) was added to block mitochondrial ATP synthesis (complex V), and to force cells to use Glycolysis for ATP production. The uncoupler carbonyl cyanide 4-(trifluoromethoxy phenylhydrazone (FCCP, 2 μM) was applied to measure respiratory reserve capacity and maximal respiration. Finally, combination of rotenone plus antimycin A (0.5 μM) was added to inhibit complexes III and I, in order to completely block mitochondrial oxygen consumption. These three compounds are serially injected to measure ATP production, maximal respiration and non-mitochondrial respiration, respectively. Spare respiratory capacity is then calculated using these parameters and basal respiration.

### MitoTracker red CMXRos assay

The mitochondrial morphology was measured by using MitoTracker Red CMXRos (Molecular Probes^™^, Thermo Fisher Scientific Inc.), a red-fluorescent dye, whose accumulation is dependent upon membrane potential, stains mitochondria in live cells. A549 cells after 48 h treatment were stained at a final concentration of 100 nM for 30 min at 37°C. Red fluorescence images were acquired per slide for microscopic observation with a fluorescence microscope (EVOS^™^ Auto 2, Invitrogen, WA, USA), and fluorescence intensity was measured using Image J by observers who were blinded to the experimental groups.

### JC-1 assay

The mitochondrial membrane potential (Δψm) was measured with a commercial JC-1 kit (Beyotime, Shanghai, China) following the manufacturer’s instructions. Briefly, A549 cells were treated as indicated and stained with 10 μM of JC-1 according to the manufacturer instructions (Beyotime, Shanghai, China). Then, the intensity of red (aggregate JC-1)/green (monomeric JC-1) fluorescence was detected with an inverted microscope (Olympus, BX53, Japan) and the ratio of the red: green fluorescence was proportional to the mitochondrial membrane potential.

### mtDNA copy number

mtDNA copy number was measured using a method described previously [50]. Briefly, total intracellular DNA was isolated from A549 cells using a FlexiGene DNA Kit (Qiagen, Hilden, Germany) following the manufacturer’s protocol. DNA was quantified spectrophotometrically (260 nm) and subjected to Real-time PCR using a CFX Connect™ Real-Time PCR Detection System (Bio-Rad Laboratories Inc., USA). NADH dehydrogenase subunit 1 (ND1) was on behalf of mtDNA amplification and lipoprotein lipase (LPL) was on behalf of an internal control. Compared the relative amount of mtDNA with nuclear DNA (nDNA) copy numbers, the ddCT (ND1/LPL) represented the mtDNA copy number in a cell. All primers were designed and synthetized by Sangon Biotech Co., Ltd. (Shanghai, China).

### SA-β-Gal activity measurement

Senescence-associated β-galactosidase (SA-β-Gal) activity was measured with a commercial kit (Genmed, MA, USA) according to the manufacturer’s instructions. Briefly, cultured cells treated as indicated were washed in Reagent A and fixed with Regent B for 5 min at room temperature. After being washed twice with Regent C, the cells were then incubated overnight at 37°C in freshly prepared staining buffer. At the end of incubation, the cells were microscopically examined at 200× magnification (Leica, Germany).

### Real-time PCR

Total RNA was extracted from A549 cells using Trizol reagent (Invitrogen Corporation, CA, USA) followed by reverse transcription using a PrimeScript™ RT MasterMix Kit (Takara, Shiga, Japan). Real-time PCR was performed with the above cDNA using SYBR Green Fast qPCR mix (Takara) with an iCyler iQ Real-time PCR Detection System (Bio-Rad Laboratories Inc., USA). 18S was used as a housekeeping gene. Experimental cycle threshold values were normalized to 18S, and relative mRNA expression was calculated versus a control sample (the primers used are listed in Table 1).

**Table 1:** Primers sets used for real-time PCR.

**Gene**	**Sense primer(5′-3′)**	**Antisense pimer(5′-3′)**
human p21	TGTCCGTCAGAACCCATGC	AAAGTCGAAGTTCCATCGCTC
human p67phox	CCAGAAGCATTAACCGAGACAA	CCTCGAAGCTGAATCAAGGC
human SIRT1	TAGCCTTGTCAGATAAGGAAGGA	ACAGCTTCACAGTCAACTTTGT
human OPA1	TGTGAGGTCTGCCAGTCTTTA	TGTCCTTAATTGGGGTCGTTG
human IL-6	CTGCAAGAGACTTCCATCCAG	AGTGGTATAGACAGGTCTGTTGG
human IL-8	CTGGCCGTGGCTCTCTTG	CCTTGGCAAAACTGCACCTT
human MMP2	AGTCTGAAGAGCGTGAAG	CCAGGTAGGAGTGAGAATG
human COXI	GATTTTTCGGTCACCCTGAAG	CTCAGACCATACCTATGTATC
human COXII	CTATCCTGCCCGCCATCATC	GATTAGTCCGCCGTAGTCGG
human COXIII	CACATCCGTATTACTCGCATC	GAAGTACTCTGAGGCTTGTAG
human Cyto b	CAAACTAGGAGGCGTCCTTG	CTGGTTGTCCTCCGATTCAG
human ATPase 6	CTTGGTCCCATCTACCTCCTC	CCCCGAACATTGCCTGGTT
human ND1	CATTCCTAATGCTTACCGAACG	GTAGAGGGTGATGGTAGATGTG
human LPL	AAGAGAGAACCAGACTCCAATG	TATTGGTCAGACTTCCTGCAAT
18s	GCAATTATTCCCCATGAACG	GGCCTCACTAAACCATCCAA

### Western blot

Western blot analysis was performed as described previously [51]. Briefly, the A549 cells were lysed in ice-cold radioimmune precipitation assay (RIPA) lysis buffer with phosphatase inhibitor (Roche) and centrifuged to obtain supernatants. Protein concentration was measured by bicinchoninic acid (BCA). Equal amounts of protein were loaded into each lane onto 10% SDS-polyacrylamide gels, transferred to PVDF membranes and incubated with appropriate primary antibodies overnight at 4°C. After reacting with HRP-labeled secondary antibodies, the immunoreactive bands were visualized using an ECL chemiluminescent kit and then scanned with Tanon-5200 (Tanon). The results were analyzed by Image J.

### Data and statistical analysis

Data analysis was performed using SigmaPlot 12.5 (Systat Software, Inc., Chicago, IL, USA), and expressed as means ± SEM. One-way ANOVA was performed for comparisons among multiple groups. *P* < 0.05 was considered statistically significant.

## Supplementary Material

Supplementary Figures
