# Supplementary material for: Hydrogen sulfide attenuates mitochondrial dysfunction-induced cellular senescence and apoptosis in alveolar epithelial cells by upregulating sirtuin 1
Source: Aging (Albany NY). 2019 Dec 23;11(24):11844–64. doi: 10.18632/aging.102454 (PMC6949053; doi:10.18632/aging.102454)
Supplement: Supplementary Figures [file aging-11-102454-s001..pdf]

SUPPLEMENTARY FIGURES

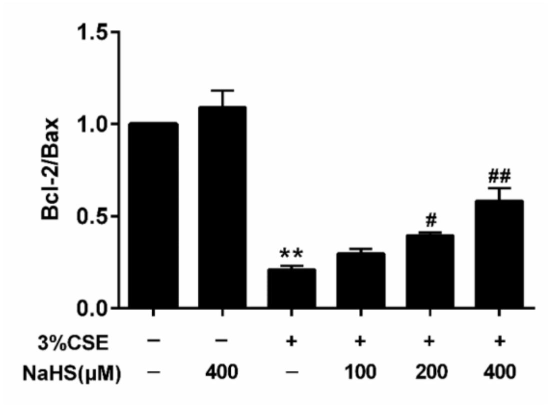

Supplementary Figure 1. Effects of NaHS on the ratio of Bcl-2 to Bax in CSE-stimulated A549 cells. A549 cells were cultured with and without 3% CSE and/or 100, 200, or 400μM NaHS for 48 h. The ratio of Bax/Bcl-2. **P<0.01, significantly different from control cells [3%CSE (-) and NaHS (-)]; #P<0.05, ##P<0.01, significantly different from cells treated with 3%CSE only.

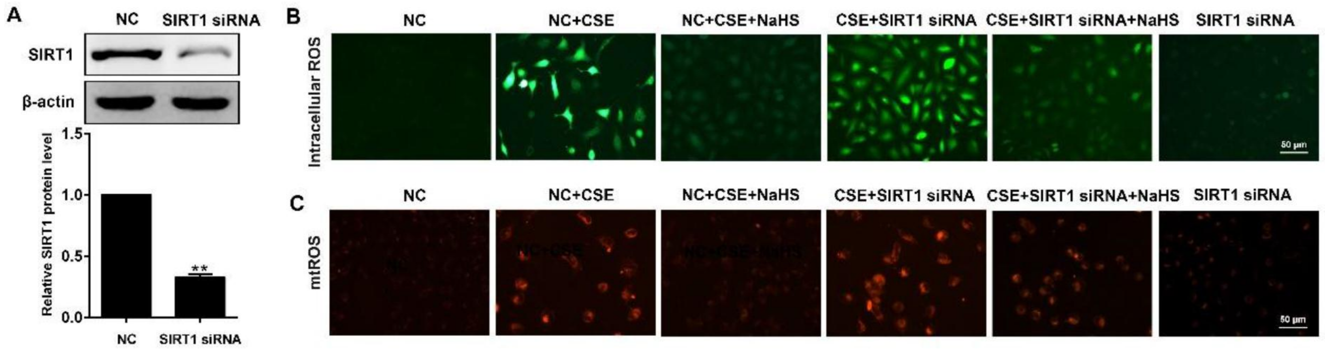

Supplementary Figure 2. Effects of NaHS on oxidative stress in CSE-stimulated A549 cells after SIRT1 was silenced. (**A**) After SIRT1 siRNA or NC siRNA was transfected into A549 cells for 24 h, SIRT1 protein expression was measured with Western blot. After SIRT1 siRNA or NC siRNA was transfected into A549 cells for 24 h, cells were treated with 3% CSE and NaHS (400μM) for 48 h. (**B**) Generation of intracellular ROS was determined by the ROS Assay Kit. (**C**) Generation of mtROS was determined by the MitoSOX^{TM} Red Assay Kit.

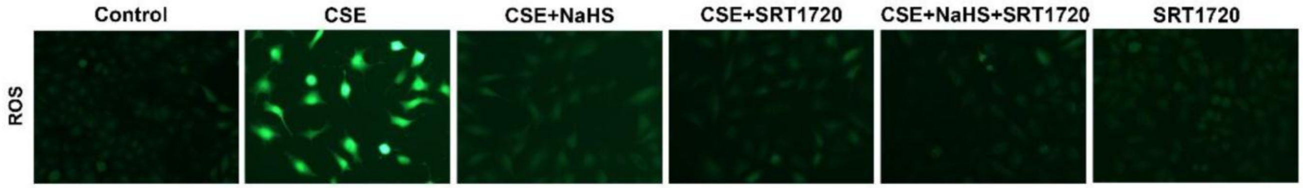

Supplementary Figure 3. SIRT1 activator (SRT1720) inhibits CSE-induced oxidative stress in epithelial A549 cells. A549 cells were treated with 400μM NaHS with or without SRT1720 (4μM) in the presence of 3%CSE for 48 h. Generation of intracellular ROS was determined by the ROS Assay Kit.

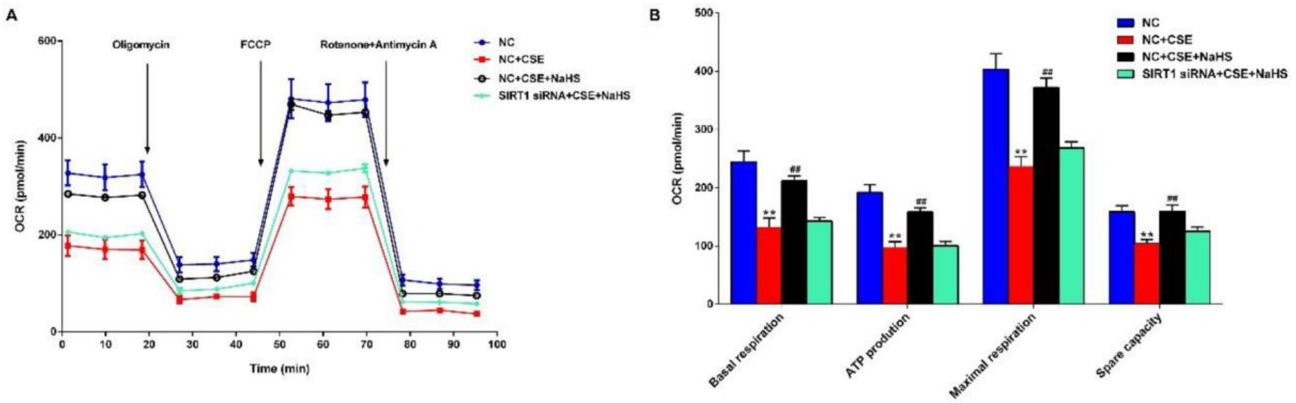

Supplementary Figure 4. Effects of NaHS on mitochondrial function in CSE-stimulated A549 cells after SIRT1 was silenced. After SIRT1 siRNA or NC siRNA was transfected into A549 cells for 24 h, cells were treated with 3% CSE and NaHS (400μM) for 48 h. (**A**) The bioenergetic profiles of A549 cells were measured by a Seahorse Extracellular Flux Analyzer, OCR in cells treated with oligomycin, FCCP, and rotenone & Antimycin A. (**B**) Quantitative analysis of basal respiration, ATP production, maximal respiratory and spare capacity is shown. $^{**}P$<0.05, significantly different from control cells [with NC siRNA transfection]; $^{\#\#}P$<0.01, significantly different from cells treated with 3%CSE only [with NC siRNA transfection].

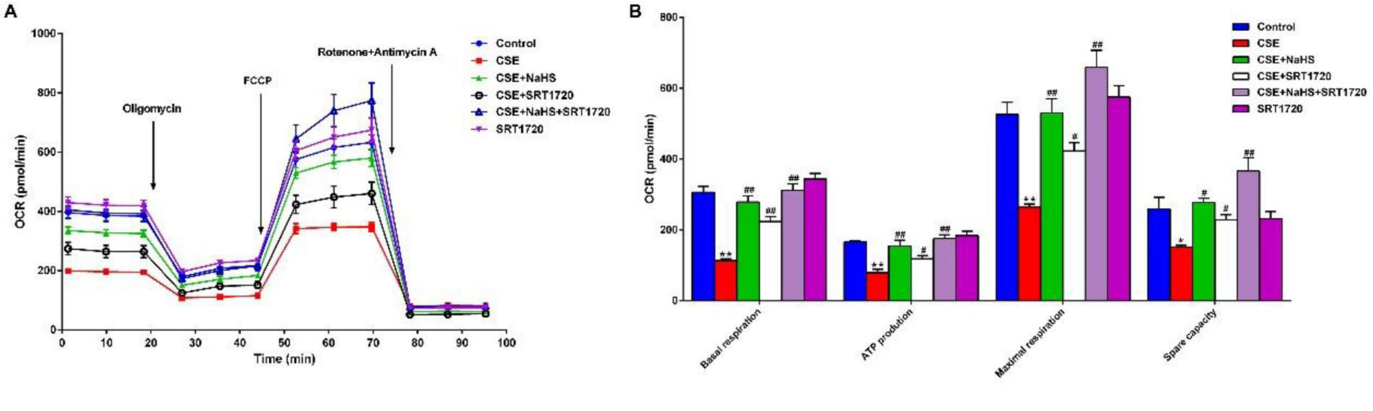

Supplementary Figure 5. SIRT1 activator (SRT1720) inhibits CSE-induced mitochondrial dysfunction in epithelial A549 cells. A549 cells were treated with 400μM NaHS with or without SRT1720 (4μM) in the presence of 3%CSE for 48 h. (**A**) The bioenergetic profiles of A549 cells were measured by a Seahorse Extracellular Flux Analyzer, OCR in cells treated with oligomycin, FCCP, and rotenone & Antimycin A. (**B**) Quantitative analysis of basal respiration, ATP production, maximal respiratory and spare capacity is shown. $^{*}P$<0.05, $^{**}P$<0.01, significantly different from control cells [3%CSE (-), NaHS (-) and SRT1720 (-)]; $^{\#}P$<0.05, $^{\#\#}P$<0.01, significantly different from cells treated with 3%CSE only.

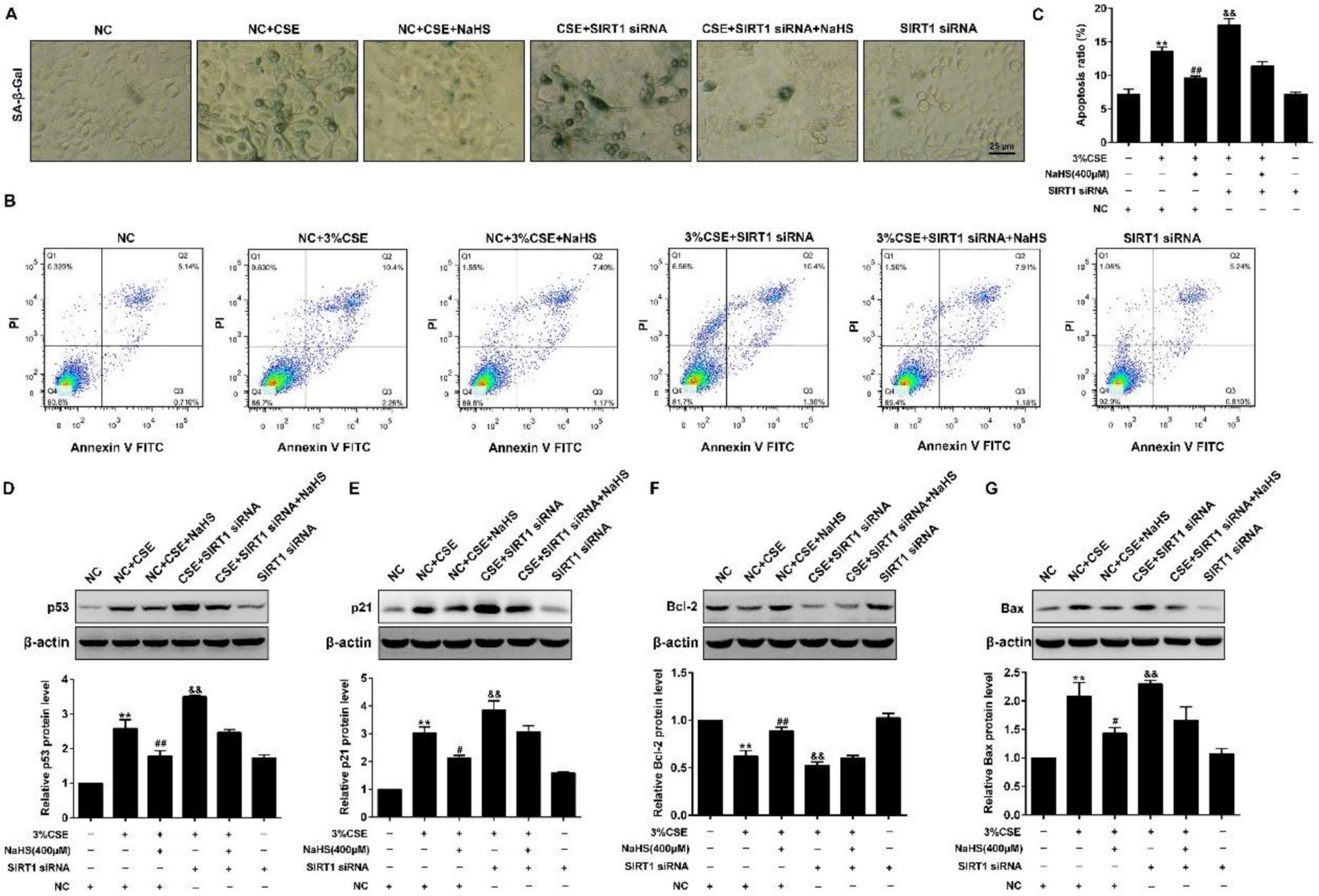

Supplementary Figure 6. Effects of NaHS on cellular senescence and apoptosis in CSE-stimulated A549 cells after SIRT1 was silenced. After SIRT1 siRNA or NC siRNA was transfected into A549 cells for 24 h, cells were treated with 3% CSE and NaHS (400μM) for 48 h. (**A**) Cell senescence was performed by examining the the SA–β-gal activity. (**B**) The cells were double-stained with Annexin V-FITC and PI, and then the cellular apoptosis was determined by flow cytometry. (**C**) The ratio of apoptotic cells was statistically analyzed. (**D**–**G**) Western blot was used to analyze the protein levels of p53, p21, Bcl-2 and Bax. $^{**}P<0.05$, significantly different from control cells (with NC siRNA transfection); $^{\#}P<0.05$, $^{\#\#}P<0.01$, significantly different from cells treated with 3%CSE only (with NC siRNA transfection). $^{\&\&}P<0.01$, significantly different from untreated cells (with SIRT1 siRNA transfection).

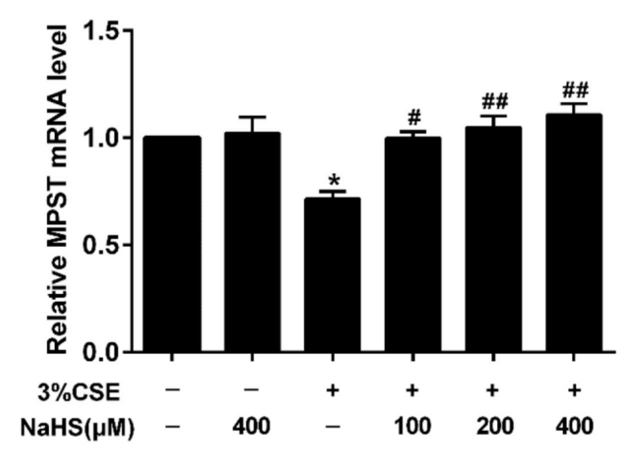

Supplementary Figure 7. Effects of NaHS on the mRNA level of MPST in CSE-stimulated A549 cells. A549 cells were cultured with and without 3% CSE and/or 100, 200, or 400μM NaHS for 48 h. Real-time PCR was performed to examine the MPST mRNA level. $^{*}P$<0.05, significantly different from control cells [3%CSE (-) and NaHS (-)]; $^{\#}P$<0.05, $^{\#\#}P$<0.01, significantly different from cells treated with 3%CSE only.
